# 

**Published:** 1901-04-13

**Authors:** 


### NOTICE TO CORRESPONDENTS.

All MSS., Letters, Books for Review, and other matters intended for the Editor should be addressed The Editor, "The Hospital," The Hospital Building, 28 & 29 Southampton Street, Strand, W.C.

The Editor cannot undertake to return rejected MS., even when accompanied by stamped directed envelope.

**Notice to Advertisers and Subscribers.**

All Advertisements, Orders for Copies of The Hospital, and Business Communications should be addressed to **THE MANAGER (Not to the Editor)**, The Hospital, 28 & 29 Southampton Street, Strand, London, W.C.

The Cost of Subscription, post free, is as follows:—Per week, 2½d.; per quarter, 2s. 8d.; per half-year, 5s. 3d.; per annum, 10s. 6d. Foreign:—Per annum, 15s. 2d., payable, in all cases, in advance.

**NOTICE.—Vol. XXIX. of "The Hospital" (October 1900, to March, 1901), in handsome cloth binding will shortly be on sale at the Office of this Journ al price, 7s. 6d. Cases for binding the Numbers contained in this Volume 1s. 6d. Index to Vol. XXIX., 2½d., post free.**

**The Monthly Part for April is now ready. Price 6d., by post 8d.**

### SCRAPS AND GLEANINGS.

THE wards of the National Hospital for Diseases of the Heart, Soho Square, will be reopened for in-patients the week after Easter.

LADY EDWINA ROBERTS, who is collecting comforts for the soldiers at the front, has sent to the Officer Commanding at Pretoria 4,000 cakes of Bovril Chocolate for general distribution.

HER MAJESTY, Queen Alexandra, has graciously consented to continue her patronage to the Ladies' Association of the Eastern Counties' Asylum for Idiots, Imbeciles, and the Feeble-minded.

DURING 1900 the extern cases treated by the Belfast Royal Victoria Hospital numbered 25,350, and the daily average of patients in the institution was 161. The total expenditure during the year was £12,914, the income being £13,980, of which £3,881 comes as bequests.

DURING the last twenty years £17,890 has been spent on the improvements which have been made at the York County Hospital, the result of which expenditure has been to bring the institution up to date and in accordance with the present requirements of the population. The report of this year's working shows an adverse balance in the accounts of £2,453, and more than one of the speakers at the annual meeting suggested as a remedy that the number of patients would have to be restricted.

### THE STUDENT OF MEDICINE.

### APPOINTMENTS.

**George Ashton**, M.B., Ch.B.Vict., M.R.C.S.Eng., L.R.C.P.-Lond., appointed Senior House Surgeon to the Bolton Infirmary. **Wm. Edmunds**, M.A., M C.Cantab., F.R.C.S., appointed Honorary Surgeon to the Tottenham Hospital. **A. Leonard Fuller**, M.R.C.S.Eng., L.R.C.P. Lond., Assistant Anæsthetist to the Royal United Hospital, Bath. **H. W. Garden**, M.B., Ch.B.Aberd. appointed Medical Officer to the Tati Concessions, Francistown, Rhodesia. **John A. Hogg**, L.R.C.P.Lond., M.R.C.S.Eng., appointed Medical Officer of Health to the Shardlow Rural District Council. **B. H. Kingsford**, M.B.Lond., M.R.C.S.Eng., appointed Certifying Factory Surgeon for the Woking District of Surrey. **R. A. Needham**, B.Sc., M.B., Ch.B., appointed Junior Demonstrator of Anatomy at Owens College, Manchester. **W. E. Nelson**, M.R.C.S., L.R.C.P., appointed District Medical Officer of the Stratford-on-Avon Union. **A. J. Whiting**, M.D.Edin., M.R.C.P.Lond., appointed Honorary Assistant Physician to the Tottenham Hospital.

